# Peak Match Demands in Young Basketball Players: Approach and Applications

**DOI:** 10.3390/ijerph17072256

**Published:** 2020-03-27

**Authors:** Enrique Alonso, Nicolas Miranda, Shaoliang Zhang, Carlos Sosa, Juan Trapero, Jorge Lorenzo, Alberto Lorenzo

**Affiliations:** 1 Faculty of Sports Sciences, European University of Madrid, 28670 Villaviciosa de Odón, Spain; jthnut@yahoo.es; 2 Catapult Sports, Melbourne 3181, Australia; Nicolas.Miranda@catapultsports.com; 3 Division of Sports Science & Physical Education, Tsinghua University, Beijing 100084, China; zsl.inef@gmail.com; 4 Faculty of Physiotherapy, European University of Madrid, 28670 Madrid, Spain; carlos.sosa@universidadeuropea.es; 5 Polytechnic University of Madrid, 28031 Madrid, Spain; alberto.lorenzo@upm.es

**Keywords:** basketball, worst case scenario (WCS), most intense passages, most demanding periods, peak demands (PD), performance

## Abstract

Background: The aim of this study is to describe the peak match demands and compare them with average demands in basketball players, from an external load point of view, using different time windows. Another objective is to determine whether there are differences between positions and to provide an approach for practical applications. Methods: During this observational study, each player wore a micro technology device. We collected data from 12 male basketball players (mean ± SD: age 17.56 ± 0.67 years, height 196.17 ± 6.71 cm, body mass 90.83 ± 11.16 kg) during eight games. We analyzed intervals for different time windows using rolling averages (ROLL) to determine the peak match demands for Player Load. A separate one-way analysis of variance (ANOVA) was used to identify statistically significant differences between playing positions across different intense periods. Results: Separate one-way ANOVAs revealed statistically significant differences between 1 min, 5 min, 10 min, and full game periods for Player Load, F (3,168) = 231.80, η_p_^2^ = 0.76, large, *p* < 0.001. It is worth noting that guards produced a statistically significantly higher Player Load in 5 min (*p* < 0.01, η_p_^2^ = −0.69, moderate), 10 min (*p* < 0.001, η_p_^2^ = −0.90, moderate), and full game (*p* < 0.001, η_p_^2^ = −0.96, moderate) periods than forwards. Conclusions: The main finding is that there are significant differences between the most intense moments of a game and the average demands. This means that understanding game demands using averages drastically underestimates the peak demands of the game. This approach helps coaches and fitness coaches to prepare athletes for the most demanding periods of the game and present potential practical applications that could be implemented during training and rehabilitation sessions.

## 1. Introduction

Basketball is an intermittent high-intensity sport where the majority of play time is spent in walking (66%) and standing activities [1]. These aerobic requirements interchange with anaerobic demands, where continuous changes of direction, jumps, sprints, accelerations, decelerations, contacts and specific skills are presented [2,3,4]. In recent years, there has been an increased interest around the use of wearable microsensor technology, such as accelerometers, gyroscopes and magnetometers to measure the workload in indoor team sports [5,6,7,8,9,10,11,12]. A main purpose in training is to prescribe the workload to develop a player’s physical performance and induce specific physiological adaptations [13,14] while avoiding overtraining and injury during the competitive season [14]. The purpose and value application of accelerometer technology is the ability to quantify external load during practices, games and rehabilitation. This provides quantitative information that can be used in the design of progressive activities to prepare the player for the demands of the game and provide objective data in return to train (RTT) and return to play (RTP) decisions [15,16,17,18,19].

Understanding game demands using averages drastically underestimates the most demanding periods of play and shows only a basic approach to staff for planning training sessions [20]. There are different methods used to determine the most intense moments. Recent studies suggest that rolling average (ROLL) is more accurate than the FIXED method [20,21]. “Most demanding periods”, “most demanding passages”, “most intense periods” and “worst case scenario (WCS)” tend to be the most common terms that researchers use to refer to the peak match demands [20,21,22,23,24,25,26,27,28]. This topic has been previously investigated in other sports such as rugby league [29], rugby union [22,26,30,31], soccer [23,25,32,33,34], mixed martial arts [27] or Australian football [21], but to our knowledge, few studies have reported information about WCS in basketball [28]. For the remainder of this article, we will refer to them as peak demands (PD).

PD and the time before and after these periods could be related with determinant moments of the match [30]. To optimally prepare athletes for the demands of competition, it is important that they are trained to endure the most demanding periods of the game and not just the average demands [20,28]. This includes highly variable, spontaneous and unanticipated technical and tactical movements reflecting the unpredictable nature of the sport. 

The information about PD offers a different insight into game demands and might present potential applications that could be implemented during training and rehabilitation sessions. The main goal of this research was to describe the most intense moments and compare them with average demands in youth basketball players, from an external load point of view using different time windows. Another objective is to determine if there are differences between positions and provide an approach for practical application. 

## 2. Materials and Methods 

### 2.1. Sample

We collected data from 12 male players (mean ± SD: age 17.56 ± 0.67 years, height 196.17 ± 6.71 cm, body mass 90.83 ± 11.16 kg) at international competitive level [35] during eight official games. 

Exclusion criteria were players that sustained an injury during a game, that accumulated less than four games or less than 8 min per game of active time on court. Participants and their parents or legal guardians were informed of the aims, risks, and benefits of the study before signing written consent to allow the collection of data for scientific purposes. The study was approved by an ethics committee (CIPI/18/195), and dissertation approval was granted by the involved basketball club; the study conformed to the Declaration of Helsinki [36].

### 2.2. Study Design

This observational study was conducted during eight games of the 2018–2019 competitive basketball season (U18 league). External workload was measured via a 100 Hz tri-axial accelerometer. The variable recorded was Player Load™ (T6, Catapult Sports, Australia), which considers the instantaneous rate of change of acceleration in three different planes (x-, y-, and z-axis) measured in arbitrary units (au) ((12,19)). This parameter has been used in several studies [5,8,9,11,12,28,29,37] and the formula is √ (ay1 − ay-1)2 + √ (ax1 − ax-1)2 + √ (az1 − az-1)2/100 [19].

During each game, every player wore a micro technology device (sample 100 Hz, T6, Catapult Sports, Australia) in a pocket under his playing jersey (on the upper thoracic spine between the scapula), which previous studies have suggested to be an accurate location [11]. The reliability of this technology has previously been shown to be acceptable for measuring external parameters in team sports [5,7,9,12,37,38].

### 2.3. Procedures

To determine the full game demands, the initial processing of the data was done with Catapult’s proprietary software, Openfield (version 1.22.0) (Catapult Sports, Melbourne, Australia). We exported the data to a custom-built Microsoft Excel spreadsheet for further analysis. The warmup, time spent on the bench, 1 minute breaks and halftime were excluded from the analysis.

To determine the most intense intervals, first, we extracted the cumulative data in each 30 second interval for each player. After that, we exported the data to a custom-built Microsoft Excel spreadsheet for further analysis; lastly, we analyzed different intervals using rolling averages (ROLL). This procedure is more accurate in determining the most intense periods than the FIXED method [16] and has been previously used in different sports such as rugby union [7,20,31], soccer [23,24] and Australian football [21]. Rolling series were stopped at the end of each quarter. Thus, rolling started at the beginning of each quarter and stopped at the end of the same quarter. For each game, the peak match demand for each player was determined. Peak workload intensities across each time window were expressed as PL·min-1 and total Player Load in absolute values. The method of understanding volume and intensity was providing an absolute Player Load (volume) and relative Player Load (intensity) given that the rate of accumulation for the given parameter is relative.

### 2.4. Statistical Analysis

Mean ± standard deviation was calculated for all workload variables. The normality of data distribution and sphericity were confirmed using the Shapiro–Wilk statistic and Levene’s test for equality of variances, and thus parametric analyses were used. A separate one-way analysis of variance (ANOVA) was used to identify statistically significant differences between different intense periods (1 min, 5 min, 10 min and full game). 

Bonferroni was used to determine significance. The effect size for each ANOVA was determined using partial eta squared (η_p_^2^) and was classified as follows: no effect = 0 to 0.039, minimum = 0.04 to 0.24, moderate = 0.25 to 0.63, and large = ≥0.64 [39]

Effect sizes for all pairwise comparisons were determined using Cohen’s d with 95% confidence intervals. Cohen’s d was interpreted as follows: trivial = 0 to 0.19, small = 0.2 to 0.59, moderate = 0.6 to 1.19, large = 1.2 to 1.99, very large = 2.0 to 3.99, and nearly perfect = ≥4.0 [39]. Effect size and subsequent 90% confidence intervals (90% CI) were calculated and visualized in the “effsize” and “ggstatsplot” package, with all analyses being undertaken using R version 3.2.5 (R Core Team, Vienna, Austria, 2015). Statistical significance was set at 0.05.

## 3. Results

**Figure 1:**
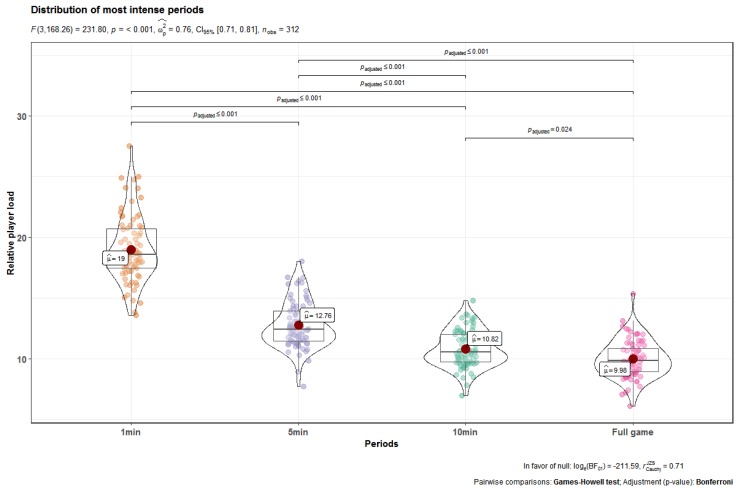
The descriptive and comparative analysis of average demands among different intense periods.

The descriptive and comparative analysis of average demands across different peak periods are presented in Figure 1. Separate one-way ANOVAs revealed statistically significant differences between 1 min, 5 min, 10 min, and full game periods for Player Load, F (3,168) = 231.80, η_p_^2^ = 0.76, large, *p* < 0.001. Post-hoc testing showed that the PL·min-1 in a 1 min window was clearly higher than in 5 min (*p* < 0.001, η_p_^2^ = 0.43, small), 10 min (*p* < 0.001, η_p_^2^ = 0.52, small), and full game (*p* < 0.001, η_p_^2^ = 0.69, moderate) periods.

The descriptive statistics of the intensity and volume for different time windows are displayed in Table 1.

The effect sizes (d) for all pairwise comparisons between conditions are shown in Table 2 and Table 3. Separate one-way ANOVAs revealed statistically significant differences between guards, forwards and centers for player load in 5 min (*p* < 0.022, η_p_^2^ =0.097, trivial), 10 min (*p* < 0.002, η_p_^2^ = 0.149, trivial), and full game (*p* < 0.017, η_p_^2^ = 0.056, trivial) periods. Post-hoc testing showed that guards produced a statistically significantly higher Player Load in 5 min (*p* < 0.01, η_p_^2^ = −0.69, moderate), 10 min (*p* < 0.001, η_p_^2^ = −0.90, moderate), and full game (*p* < 0.001, η_p_^2^ = −0.96, moderate) periods than forwards.

## 4. Discussion

The main finding was that there are differences between PD moments and game demands based on averages for every time window. This means that understanding game demands using averages drastically underestimates the peak demands of game. We should consider the way in which we understand competition demands and, consequently, whether we are preparing players to cope with peak game demands. The majority of the studies about game demands in basketball are based on averages [2,3,4,40,41]. To our knowledge, only one previous study based on one game has reported information considering the most intense passages in basketball [28]. 

Positional differences presented in the current article show that guards produce a higher Player Load during 5 min PD windows and the full game compared to forwards. This suggests that taking into consideration the specific profiles for our squad could be an efficient strategy for the individualization of training and to prepare the players for the most physically demanding passages of the game. We should understand specific profiles for our squad and prepare the players depending on team context, field role and individual characteristics.

Although real competition has a chaotic nature [17] and provides a higher stress response than a simulated context does, a common way of simulating game demands during practice sessions is in the form of small-sided games (SSGs) [42,43,44], which are useful for performing and maintaining match-specific technical and tactical features. During these type of drills, it is common to manipulate certain factors such as the number of players, work-to-rest ratios, the size of the court, the rules of the game and coach feedback, which in turn also alter the physiological and perceptual burden [42,43,45,46]. 

During the current study, we presented potential further limitations of SSGs, which might not be specific enough to address player necessities or may not provide enough stimulus to build key specific demands [47]. To the knowledge of the authors, only one article performed in soccer has analyzed the load that SSGs require compared with peak match demands [32].

A solution for this could include additional high-intensity interval training (HIIT) methodologies. Although several authors have revealed that SSGs are as efficient as HIIT in developing specific aerobic fitness for team sports [43,48], incorporating high-intensity interval training, where players are exposed to the most intense moments, might be an interesting approach to achieve individual player necessities.

Another tool to achieve the desired intensities during training sessions is modifying the rules of 5 vs. 5 games [45,46,49]. Avoiding free throws and time-out allowances modulates the physical load [45]. Additionally, it is difficult to replicate basketball game demands using half-court situations [46]. A nonstop game (clock is not stopped, no free throw after a foul, quick ball in play reposition and no time-outs) elicits a greater physiological response and fatigue than a regular stop game (the clock is stopped when the ball is out of bounds, and clock is stopped for fouls) [49]. Neither of these studies have researched whether modifying 5 vs. 5 games is sufficient to recreate the peak demands of the game. 

We propose that future research should try to understand which methodology is the most adequate to deliver a workload similar to PD passages. Practitioners should apply this depending on the context and objectives of the session.

In spite of that, the information about the most demanding moments offers a different insight into game demands and might be used to prepare for the most intense passages of matches [20,28].

### 4.1. Practical Applications

This approach presents valuable potential applications that could be used by strength and conditioning coaches. Depending on the context, these could be implemented during training sessions with unselected or fringe players or rehabilitation sessions.

#### 4.1.1. Training Sessions

One of the most common practice cues utilized by staff during training sessions is to train at “game intensities”. A main objective during practice is to prescribe the adequate load to induce specific physiological adaptations [13,15] while reducing the risk of overtraining and injury [14].

It should be borne in mind that, in some contexts, teams have congested schedules, with 2–4 games per week, and this approach may not be necessary during training sessions. Depending on the orientation, games per week, individual necessities, individual stress tolerance and objectives, coaches and strength conditioning professionals should find a way to expose players to the external peak match demands during practice sessions in order to prepare the athletes to cope with the most intense demands of the game.

#### 4.1.2. Unselected or Fringe Players

The issue of starters and benching players is of interest to fitness coaches in team sports because of differences in the training and match loads between both statuses [50,51,52,53,54,55]. Coaches and fitness coaches should concurrently consider the number of weekly games and the player status, as well as examining individual player performance and creating individualized periodization plans, increasing the total load potentially needed from bench players, especially in two-game weeks [55]. 

Furthermore, a lower number of decelerations (<16) and less distance covered (2 km) is significantly associated with injury during professional basketball games [50]. Increasing external workload when this happens may likely reduce the risk of injury [50]. The workload is drastically different between weeks [52], and athletes who sit on the bench during matches may need additional training outside of games in order to balance the workload [53] and prepare athletes to cope with the PD of the game [20,28]. 

Thus, it might be interesting to allow players who are unselected or athletes who only play for a few minutes to replicate peak game demands in order to balance their workload. One solution could be interval training, in which players interchange intensity based on average demands, with high-intensity efforts simulating the most intense external demands. In this way, players could accumulate the desired volume and be exposed to the PD of the game.

#### 4.1.3. Return to Play

In an elite environment, the return-to-sport (RTS) process is a constant learning and dynamic process which is influenced by several variables. Therefore, both the pros and cons (to the team and to the player) must be carefully considered in the decision-making process [56,57]. Before RTT and RTP, it is important that players demonstrate optimal levels of physical capacities to perform game demands to minimize the risk of reinjury [15].

Athletes during rehabilitation are often prescribed a workload similar to a typical week, with the aim of exposing the athlete to the adequate training intensity and volume to protect them against reinjury [56]. The PD approach provides volume and intensity information that could be useful to prepare specific progressions, where players are exposed to the most demanding passages before the RTT and RTP decision-making process.

There are several phases during RTS, each of which has different objectives [15,17]. The final stage of RTP is to ensure that the player is ready to endure full-team training and cope with the game’s demands [15]. Drills should include the most realistic physiological, physical and mental requirements to prepare players with a competitive orientation [57]. The goals of this are to expose the player to preinjury weekly training demands (volume and intensity), and drills designed to recreate PD periods are introduced (physical demands/high chaos) [17].

Players should be exposed to high-intensity activities before RTT and RTP to increase the load on the injured zone, to improve physical performance [56] and to provide other valuable objective information regarding the RTS decision-making process.

### 4.2. Limitations of the Study

A significant limitation we faced during the current study is the data treatment using the ROLL method. The cumulative data in every 30 second interval for each player arise from a first FIXED method, where moving averages throughout the 30 second fixed-period data series are used. This could lead to similar problems to the FIXED method itself; i.e., underestimating the peak magnitude and the difference between peak and subsequent periods. However, for the purpose of this study, and given the limitation we faced with bulk raw 10 Hz data extraction, we decided to go for the shortest time frame we had available (30 s windows). The rolling method with a sample frequency of 10 Hz must be considered in future studies to ensure more accurate analysis. Furthermore, the methodology and main references are taken from studies carried out with GPS while this study has been carried out with accelerometers, which have been found to have higher sensibility with shorter epoch lengths.

Another potential limitation of the current study is the sample size, which used a small exclusive sample. Nevertheless, the study results are unique to U18 basketball players of this level, and this should be considered. This study provides information about the most intense moments from a volume and intensity perspective, but data about density is not presented. For a better understanding of the game’s demands, it would be interesting to know the work/rest ratio, frequency of the PD and what happens before and after these most demanding passages. Furthermore, more experience and studies are necessary to prove the practical applications from the perspective of the most intense demands. Furthermore, it is also necessary to describe the PD from an internal point of view, to understand individual physiological differences.

It is important to determine the drastic differences between the team’s technical levels: seven out of eight games were won by 30 points or more, and just one was won by three points. Furthermore, this research only investigates the most intense demands from a one-parameter point of view. We should determine which variables are adequate to describe the most demanding passages from a more comprehensive perspective.

## 5. Conclusions

The main finding of this study is that there are important differences between the most intense moments of a game and the game’s demands based on averages.

This means that understanding game demands using averages drastically underestimates the most intense moments. To optimally prepare athletes for the PD periods, it is important that they are trained to endure the specific demands of these passages of the match and not just average demands.

Furthermore, we should consider the differences between positions in order to prepare according to specific demands of each role. We should aim to understand specific profiles for our squad and prepare the players depending on the team context, field role and individual characteristics.

Further studies with robust methodologies including a larger sample, raw data extraction and the analysis of different parameters are recommended by the authors of this study. In this study, we present a practical approach which could be helpful during the design of training sessions, especially for bench/fringe players or during rehabilitation sessions, as well as for future research.

## Figures and Tables

**Table 1:** The descriptive analysis of volume (Player Load) and intensity (PL·min-1) among different time windows.

Window	PL·min-1		Total Player Load	
	Mean	SD	Mean	SD
1 min	19.15	±2.74	19.15	±2.74
5 min	12.76	±1.91	63.79	±9.55
10 min	10.82	±1.50	108.16	±15.04
Full Game	7.53	±1.53	370.67	±105.85

**Table 2:** The descriptive analysis (mean ± SD) of different time windows for different playing positions.

Window	Guard	Forward	Center	F	Sig.	Partial Eta Squared
1 min	19.92 ± 2.97	18.47 ± 2.47	18.45 ± 2.52	2.708	0.171	0.067
5 min	13.49 ± 2.13	12.15 ± 1.75	12.59 ± 1.44	4.036	0.022	0.097
10 min	11.5 ± 1.52	10.18 ± 1.44	10.77 ± 1.14	6.542	0.002	0.149
Full Game	8.3 ± 1.4	7.53 ± 1.56	8.21 ± 1.6	2.206	0.017	0.056

**Table 3:** Effect sizes (Cohen’s d with 95% confidence intervals) for pairwise comparisons between guards, forwards, and centers for player load in male U18 basketball players.

	Guards vs. Forwards			Forwards vs. Centers			Guards vs. Centers		
Time Window	Cohen’s d	Descriptor	*p*-Value	Cohen’s d	Descriptor	*p*-Value	Cohen’s d	Descriptor	*p*-Value
1 min	−0.53 (−1.06, −0.01)	*Small*	0.05	−0.01 (−0.60, 0.58)	*Trivial*	0.97	−0.53(−1.13, 0.08)	*Small*	0.08
5 min	−0.69 (−1.23, −0.15)	*Moderate*	<0.01	0.27 (−0.32, 0.86)	*Small*	0.37	−0.48(−1.08, 0.13)	*Small*	0.11
10 min	−0.90 (−1.44, −0.35)	*Moderate*	<0.001	0.45 (−0.15, 1.04)	*Small*	0.14	−0.53(−1.13, 0.08)	*Small*	0.08
Full Game	−0.96 (−1.51, −0.41)	*Moderate*	<0.001	0.53 (−0.07, 1.13)	*Small*	0.08	−0.46(−1.06, 0.14)	*Small*	0.12
